# Global variation of soil microbial carbon-use efficiency in relation to growth temperature and substrate supply

**DOI:** 10.1038/s41598-019-42145-6

**Published:** 2019-04-04

**Authors:** Yang Qiao, Jing Wang, Guopeng Liang, Zhenggang Du, Jian Zhou, Chen Zhu, Kun Huang, Xuhui Zhou, Yiqi Luo, Liming Yan, Jianyang Xia

**Affiliations:** 1 Zhejiang Tiantong Forest Ecosystem National Observation and Research Station, Center for Global Change and Ecological Forecasting, School of Ecological and Environmental Sciences, East China Normal University, Shanghai, 200062 China; 2 Center for Ecosystem Science and Society, Northern Arizona University, Arizona, Flagstaff, AZ 86011 USA

## Abstract

Soil microbial carbon-use efficiency (CUE), which is defined as the ratio of growth over C uptake, is commonly assumed as a constant or estimated by a temperature-dependent function in current microbial-explicit soil carbon (C) models. The temperature-dependent function (i.e., *CUE* = *CUE*_0_ + *m* × (*T* − 20)) simulates the dynamic CUE based on the specific CUE at a given reference temperature (i.e., CUE_0_) and a temperature response coefficient (i.e., *m*). Here, based on 780 observations from 98 sites, we showed a divergent spatial distribution of the soil microbial CUE (0.5 ± 0.25; *mean* ± *SD*) at the global scale. Then, the key parameters *CUE*_0_ and *m* in the above equation were estimated as 0.475 and *−*0.016, respectively, based on the observations with the Markov chain Monte Carlo technique. We also found a strong dependence of microbial CUE on the type of C substrate. The multiple regression analysis showed that glucose influences the variation of measured CUE associated with the environmental factors. Overall, this study confirms the global divergence of soil microbial CUE and calls for the incorporation of C substrate beside temperature in estimating the microbial CUE in different biomes.

## Introduction

Soil microbial dynamic is a key regulator of ecosystem carbon (C) cycling because of its important role in decomposition and stabilization of soil organic carbon (SOC)^1–3^. The mechanistic understanding of soil microbial dynamics has been rapidly improved in recent decades^4^, but their numeric representation in the Earth system models (ESMs) has lagged behind^5^. Recent studies have reported that it might be reasonable to incorporate microbial processes into global C model^5–9^. For example, the simulated global SOC by the Community Land Model (CLM) has been more accurate with the implementation of microbial dynamics^7–9^. In those microbial-explicit SOC models, the soil microbial carbon-use efficiency (CUE; i.e., the ratio of growth over C uptake) is a key parameter. However, there is a lack of consensus on the CUE estimate among different models^10^. Synthesis based on observations has shown that the natural variation in soil microbial CUE is larger than aquatic, coastal and estuarine ecosystems^11^. Thus, a better understanding of the microbial CUE variability in the soil is critical for improving the simulation accuracy of the microbial-explicit SOC models.

Because CUE represents the ratio of growth to assimilation rates, differences in the temperature sensitivity of these two components causes the variations in CUE as a function of temperature change. Generally, respiration increases more than growth as a function of temperature, so CUE tends to decrease with temperature in both soil and aquatic systems^6,12–17^. This pattern has already been represented in many microbial-explicit SOC models as a linear temperature sensitivity function^5,9,10,18–20^:

1 $$CUE=CU{E}_{0}+m(T\,\mbox{--}\,{T}_{0})$$

where *CUE*_0_ is the CUE at reference temperature, *m* is the temperature response coefficient (i.e., the change in CUE per °C temperature change) and *T*_0_ is the reference temperature (usually set as 20 °C). However, the parameters in this equation are usually determined from a few observations or experiments^9,18^. Thus, it remains unclear whether the parameters of the Eq. (1) can be well constrained by the observations of CUE at the global scale.

In contrast to temperature, substrate quality and accessibility are other key factors which affect the soil microbial CUE^11,21^. As summarized by Manzoni *et al*.^11^, substrate quality regulates CUE variation through two different approaches. First, substrates with different chemical structures have to undergo different metabolic pathways to be completely decomposed. For example, substrates consist of more degradable compounds such as carbohydrates and protein could result in higher CUE than those contains more recalcitrant compounds, e.g., aliphatic, aromatic and lignin^11,22–25^. Second, microbial CUE is higher for the C substrate with a high degree of reduction (*γ*_*S*_), i.e., a measure of the chemical energy per unit mole of C^11,22,26,27^. The value of *γ*_*S*_ ranges from 1 (e.g., oxalate) to 8 (e.g., methane) and is approximately equal to 4.2 in the microbial biomass^11,27^. Hence, the microbial CUE is mainly limited by energy when the *γ*_*S*_ of substrate is <4.2, but reaches the maximum for substrates with *γ*_*S*_ > 4.2^11,27^. Both of these two approaches highlight that substrate quality is critical in affecting the microbial CUE variability in the soil. Besides the substrate quality, microbial growth is strongly related to the substrate accessibility which is collectively affected by environmental and soil conditions such as water availability^28^ and aggregates^29^.

The objective of this study is to examine the impacts of temperature and substrate type on soil microbial CUE based on observations at the global scale. A data-assimilation approach based on the Markov chain Monte Carlo (MCMC) technique is applied to constrain the parameters in the Eq. (1). Specifically, the aims of this study are to: ① constrain the key parameters in the Eq. (1) based on observations; and ② explore the dependence of soil microbial CUE upon substrate type.

## Results

### Global divergence of soil microbial CUE

**Figure 1:**
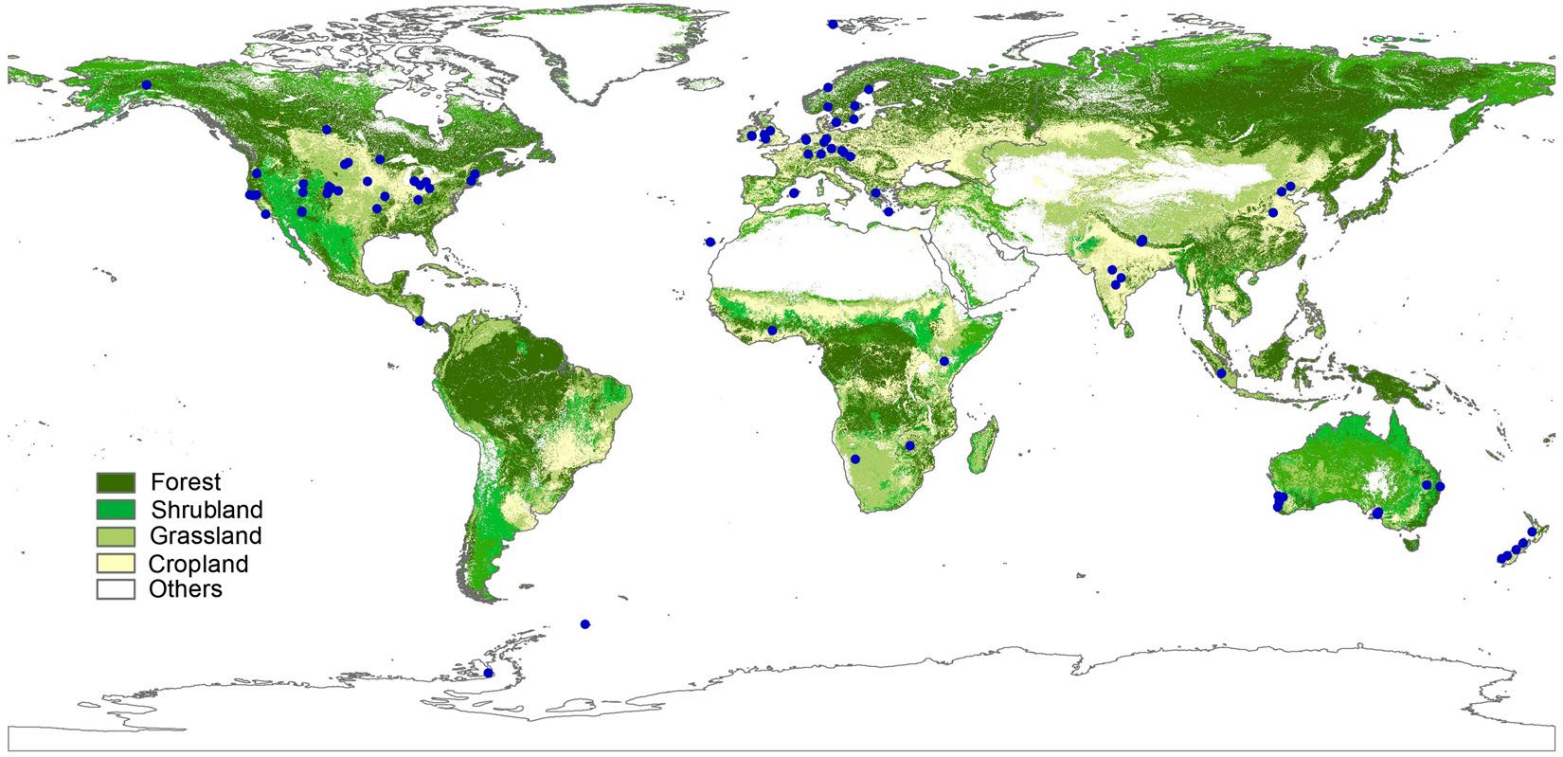
Global distribution of 98 sites all over the world. These study sites were extracted from 56 published papers. The source of spatial world map was made with Natural Earth. No permission is needed to use Natural Earth (https://www.naturalearthdata.com/about/terms-of-use/). The map was generated using Esri ArcMap 10.0 (https://www.arcgis.com).

**Figure 2:**
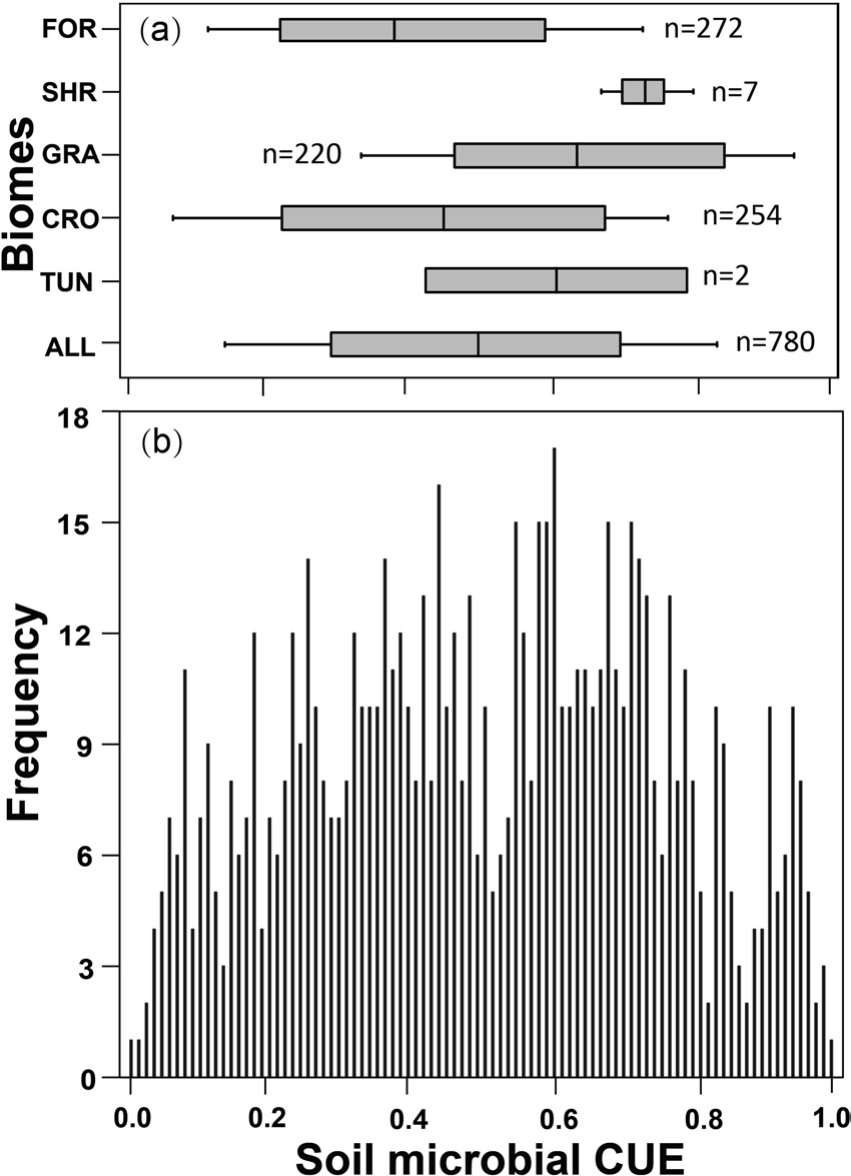
The mean CUE of different plant functional types (PFTs, **a**) and the frequency of CUE values at the global scale (**b**). FOR: Forest, SHR: Shrublands, GRA: Grasslands, CRO: Croplands, TUN: tundra, ALL: the mean CUE at the global scale.

Overall, 780 observations from 98 sites across the globe were collected in our analysis (Fig. 1, Tables S1, S2), with the measurements between the year of 1973 to 2017. The latitude of the sites ranged from 71°S to 78°N, and the longitude from 147°W to 174°E. Our data covered most terrestrial biomes, including forests, shrublands, grasslands, croplands and tundra (Fig. 2a). Globally, the mean value of CUE is 0.5 ± 0.25 (Mean ± SD) (Fig. 2b). The frequency distribution of CUE values suggests a distinct divergent distribution of CUE at the global scale. The observed CUE were highest in shrublands (0.73 ± 0.04) and grasslands (0.65 ± 0.22) but lowest in forests (0.41 ± 0.22).

### Temperature dependence of soil microbial CUE

**Figure 3:**
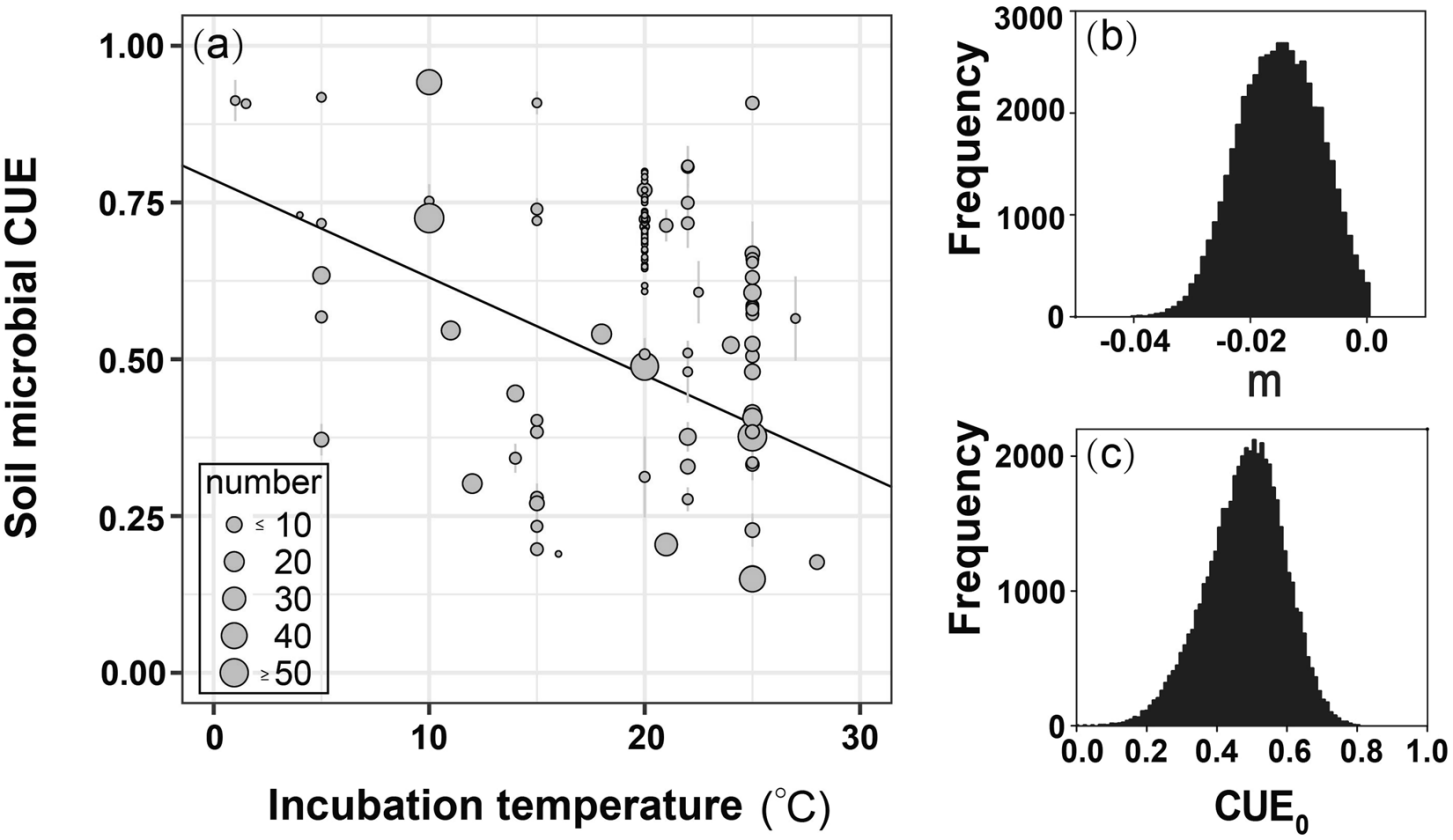
The relationship of the CUE with the corresponding incubation temperatures (**a**). The frequency of *CUE*_0_ and *m* in the Eq. (2) was shown in the panel (b,c), respectively.

The linear regression analysis detected no correlation of CUE with latitude, longitude, mean annual precipitation, mean annual temperature, soil pH or carbon to nitrogen ratio across the given sites (Fig. S1). However, a significant negative relationship was found between the CUE and the corresponding incubated temperature based on 718 values from our database (Fig. 3a). The range of the incubation temperature in this synthetic database was from 2 °C to 28 °C. The regression of CUE with temperature was matched well by Eq. (1), in which the maximum likelihood estimator of the *CUE*_0_ and *m* was 0.475 and −0.016, respectively (Fig. 3). Therefore, the temperature dependence of CUE could be better expressed as the following formula:

2 $$CUE=0.475-0.016\times (T-20)$$

### Dependence of CUE upon substrate type and degree of reduction

**Figure 4:**
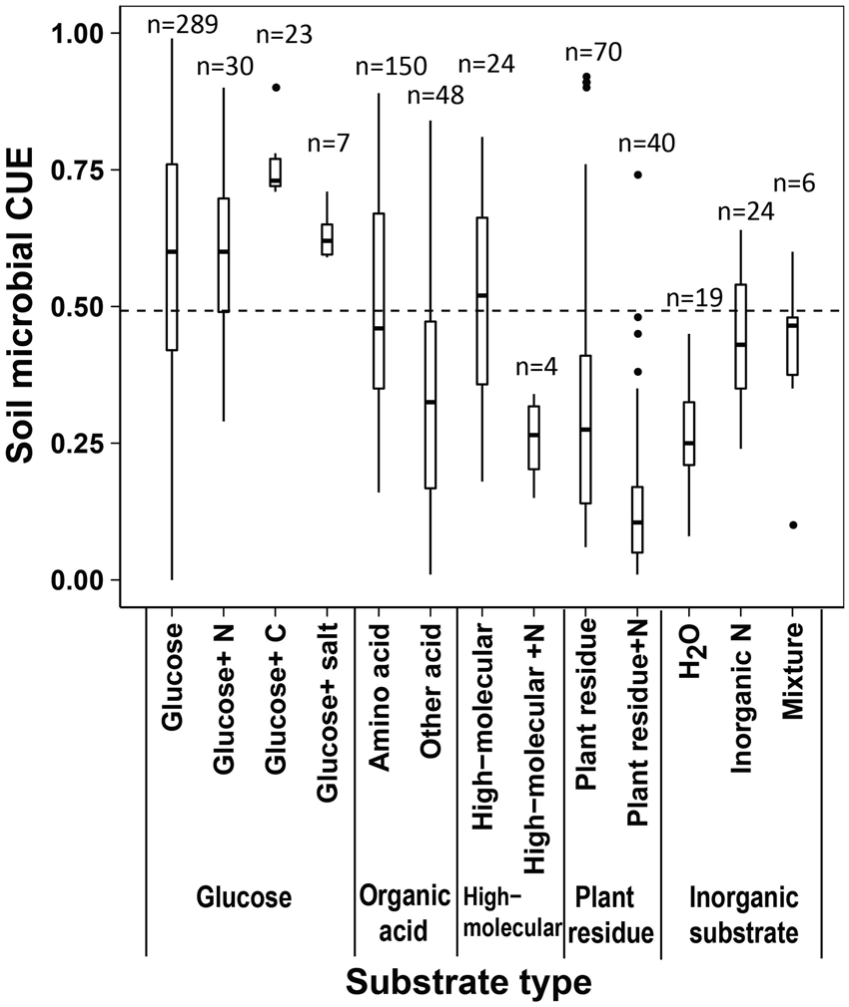
The mean CUE of main five different substrate types. (1) The substrate of glucose including glucose, sugar with inorganic N (glucose + N), glucose with other organic C (glucose + C), and glucose with inorganic salt (glucose + salt); (2) the substrate of organic acid dividing into amino acid and other acid; (3) The substrate of high-molecular including high-molecular substrate (protein, cellulose, cellobiose, plant cell walls, microbial cell walls, tri-palmitoyl-glycerol synthesis, polyhydroxybutyrate synthesis), high-molecular with inorganic N (high-molecular + N); (4) the substrate of plant residue including plant residue (a whole plant residue or plant parts (roots, leaves, stems)), plant residue with inorganic N (residue + N); (5) inorganic substrate dividing into H_2_O, inorganic N and the mixture.

**Figure 5:**
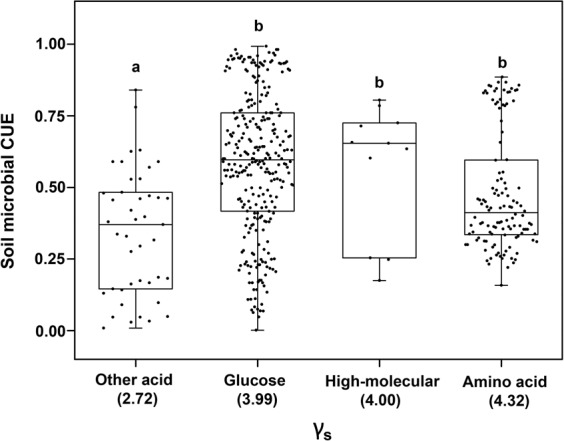
The relationship of CUE with the degree of reduction for corresponding substrate (*γ*s). Different lowercase letters represent significant difference among different substrate types (Turkey test, *P* < 0.05).

Soil microbes using different types of substrate as C sources have different CUE (Fig. 4). The microbial CUE was highest in the soils which were incubated with glucose plus organic carbon addition (0.75 ± 0.05), while was lowest in that with the residue plus N addition (0.15 ± 0.15). The mean soil microbial CUE under the addition of amino acid (0.51 ± 0.20) was comparable with that under the addition high-molecular compounds (0.51 ± 0.19). Soil microbial CUE was similar under the additions of other organic acid (0.33 ± 0.21) and plant residues (0.32 ± 0.24). Nutrient availability plays an important role in regulating microbial CUE. For example, the microbial CUE was high in soils which were incubated with glucose plus N solution (0.60 ± 0.16) or mixed inorganic solution (0.41 ± 0.17). However, for those soils with glucose addition, no positive impact of N addition on microbial CUE was detected. On the contrary, adding N lead to 51% and 53% declines of CUE under the incubation of high-molecular compounds and plant residue, respectively (Fig. 4). Based on the 462 observations of CUE with according substrate type, soil microbial CUE also varied with the *γ*_*S*_ of different substrate (Turkey test, *P* < 0.05; Fig. 5).

## Discussion

Soil microbial CUE is a commonly used parameter to quantify how soil carbon (C) is partitioned between microbial growth and respiration. The global mean CUE in our analysis (0.5) is comparable to the result of a former data-analysis across the world, where soil CUE was about 0.55^11^. These values are close to the thermodynamic maximum of metabolic efficiency (0.6)^26,27^, but is much higher than the CUE value of 0.3, which has been recommended for large-scale models by Sinsabaugh *et al*.^30^. Furthermore, the global divergence of microbial CUE with a wide range (i.e., 0.002 to 0.993) in our analysis implies that a constant CUE in biogeochemical models is inappropriate. For example, the mean CUE of soil microbes in the shrublands (0.73) is much higher than that in grasslands (0.65) or forests (0.41). The great variability of CUE at global and ecosystem-level scale emphasizes the importance of understanding the mechanisms underlying the microbial CUE variation.

Several recent modelling studies have used the Eq. (1) to simulate the variation of microbial CUE^5,31^. Among these models, however, the *CUE*_0_ is not identical and the parameter *m* ranges largely from -0.016 to 0^10,18^. In this study, we recommend an equation of *CUE* = 0.475 − 0.016 × (*T* − 20) by assimilating the observations to the Eq. (1) with the Markov chain Monte Carlo (MCMC) technique. The temperature response coefficient *m* in this formula is not 0 as in some previous studies^10,18^. Thus, the CUE is not a constant value but must changes with temperature in biogeochemical models according to this amended function. Because the rate of climate warming is faster at high than low latitudes^32^, the CUE could reduce quicker in cold than warm regions. Thus, it remains unclear how future soil C cycling in global land models will be affected if they incorporate the temperature dependent of CUE. However, it should be noted that numerous studies have shown a lack of temperature effect on CUE in the short term^33^ and a weakening temperature dependence of CUE with thermal acclimation^31,34^. Besides, the equation of CUE we recommend (i.e., *CUE* = 0.475 − 0.016 × (*T* − 20)) might not perfectly reflect the CUE value without considering other environmental factors. We further explored the CUE equation with including other environmental factors (i.e., Latitude, Substrate type, Longitude, MAP, and pH) through the multiple the stepwise regression analysis. Based on the results of multiple stepwise regression analysis (Table S3), we provided the CUE formula with all environmental factors (i.e., Temperature, Latitude, Substrate type, Longitude, MAP, and pH) included:

$$\begin{array}{rcl}{\rm{CUE}} & = & 0.319-0.025\times (T-20)+0.172\times Glucose-0.006\times Latitude\\  &  & -0.001\times Longitude+0.0002\times MAP+0.032\times pH.\end{array}$$

**Figure 6:**
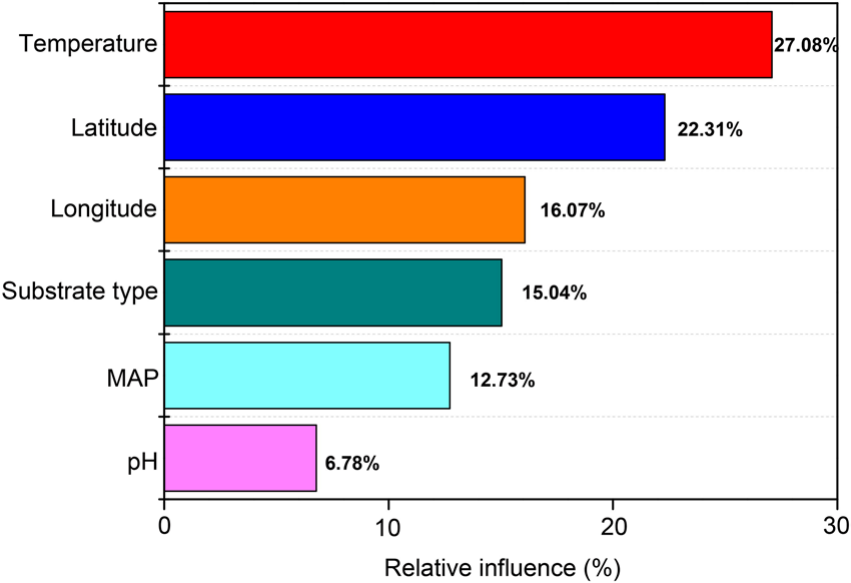
Relative influence of single factor (Latitude, Longitude, Temperature, Substrate type, MAP, pH) on CUE based on multiple stepwise regression analysis.

The selection of glucose rather than other types of C substrate suggests that glucose has a large impact on the global variation of measured CUE. We further compared the two fitted equations between data of two substrate categories (i.e., glucose and others) (Table S4). We found that the CUE_0_ was higher with adding glucose (0.491) than other types of substrate (0.319). This study also found that latitude is another important factor influencing microbial CUE. The percentage contribution of latitude reaches 22.31% when all factors (Latitude, Longitude, Temperature, Substrate type, MAP, pH) are included in the multiple stepwise regression analysis for explaining the CUE variation (Fig. 6), though there is no significant relationship between latitude and CUE in the univariate regression analysis (Fig. S1(a)). We also assessed all the environmental factors (i.e., Latitude, Longitude, Temperature, MAP, and pH) with a Principal Component Analysis (PCA) (Fig. S2).

Numerous laboratory studies and field investigations have found strong dependence of CUE upon substrate type and nutrient condition^35–37^. In this study, the soil microbes have higher CUE with the easily degradable (such as glucose, amino acid and high-molecular organic matter) than the recalcitrant (plant residues) substrates (Fig. 4). This could be due to the greater activation energies are consumed for enzyme production and excretion during the decomposition of the recalcitrant than easily degradable compounds^11^. Another potential mechanism is that the substrate accessibility of plant residues is lower than others, e.g., glucose, because of the occlusion of organic matters by aggregation^38^. Increasing nutrient availability is assumed to enhance microbial CUE mainly because of its negative effect on microbial respiration^39–41^. This assumption is partially supported by our analysis, which shows greater CUE after adding inorganic N or mixed inorganic solution than only adding water (Fig. 4). However, adding N leads to no changes and even reductions of microbial CUE in the soils which were incubated with glucose and high-molecular compounds or plant residue, respectively. The variation of microbial CUE also depends on the *γ*_*S*_ of different substrate. Our study also suggests that more degradable compounds (such as glucose) with high *γ*_*S*_ owns higher microbial CUE than the recalcitrant compounds (such as oxalate) with low *γ*_*S*_ (Figs 4 and 5). Also, because the chemical compound of litter differs greatly among plant species^42^, the substrate type must be considered to estimate the soil microbial CUE in different regions of the globe. Thus, more research efforts are still needed to explore the differences in substrate type among vegetation types and their roles in regulating the dynamics of soil microbial CUE.

In summary, this study used a data assimilation approach to constrain the temperature dependence of soil microbial CUE based on the existing observations at the global scale. The observed CUE shows a divergent distribution globally, suggesting that the parameterization of CUE is still one key challenge for the microbial-explicit soil C models. We recommend to use the constraint parameters in the Eq. (2) (i.e., CUE = 0.475 − 0.016 × (T − 20)) if soil microbial CUE is used in future global land models. However, it should be noted that tremendous additional efforts are still needed before we can correctly simulate the variations of microbial CUE in the soil. For example, as shown in this study, it is clear that the type of substrate (especially glucose) affects the simulation of soil microbial CUE. Besides, the random sampling of data has been one major challenge for almost all meta-analysis studies, because the observations of almost all variables are distributed in three hotspot regions (i.e., North America, West Europe, and China). In our study, most observations were from forest, grassland and cropland. So the data set cannot perfectly represent the distributions of global biome type and soil community in the real Earth system. We also call for more observations in other biomes such as shrubland, tundra and desert. Overall, this study highlights that the predictive ability of microbial-explicit soil C models can be enhanced by an improved understanding on the impacts of temperature and substrate type on microbial CUE.

## Methods

### Data collection

Peer-reviewed literatures related to soil microbial CUE published before July 2017 were searched using the Web of Science, according to the PRISMA (preferred reporting items for systematic reviews and meta-analyses) guidelines^43,44^ (Fig. S3). There are different methods to quantify the soil microbial CUE, so the selected studies must directly have reported the CUE or provided at least one of the following information: (1) microbial C uptake and substrate C consumption; (2) microbial C uptake and cumulative C respiration; and (3) microbial C uptake, respiration rate and the length of experiment time. The following Eqs (3–5) in this study has shown the methods for CUE calculation based on these data. Ancillary information such as latitude, longitude, mean annual temperature, mean annual precipitation, pH, C:N ratios, and substrate type were also extracted from the papers or cited papers or, in the case that it was not reported, extracted from the database at http://www.worldclim.org/ using the location information (e.g., latitude and longitude).

### Methods for data conversion

Soil microbial CUE was not directly reported but can be calculated based on its definition in many literatures. If one study reported the microbial C accumulation (Δ*MBC*) and the C substrate consumption (ΔC_substrate_), then the CUE can be calculated as:

3 $${\rm{CUE}}=\frac{{\rm{\Delta }}\mathrm{MBC}}{{{\rm{\Delta }}C}_{{\rm{substrate}}}}$$

If both the microbial C accumulation (Δ*MBC*) and cumulative microbial respiration (R_cumulative_) were reported, we calculated the CUE by using the following formula:

4 $${\rm{CUE}}=\frac{{\rm{\Delta }}\mathrm{MBC}}{{\rm{\Delta }}\mathrm{MBC}+{{\rm{R}}}_{{\rm{cumulative}}}}$$

In some studies, the cumulative microbial respiration can be obtained from the rate of respiration (*R*) over a given incubation time (*t*), so the CUE was calculated as:

5 $${\rm{CUE}}=\frac{{\rm{\Delta }}\mathrm{MBC}}{{\rm{\Delta }}\mathrm{MBC}+{\rm{R}}\times {\rm{t}}}$$

### Bayesian framework to determine CUE dependence on temperature

The Eq. (1) commonly represents the temperature dependence of CUE in most microbial-explicit SOC models^5,9,18^. The parameterization of the Eq. (1) (i.e., *CUE*_0_ and *m*) is usually arbitrary in soil enzyme driven models. For example, the *CUE*_0_ has been fixed as 0.31, 0.5 or 0.63 and parameter *m* ranges from −0.016 to 0 in previous studies^10,18^. This study employed the Bayesian probability inversion and a Markov chain Monte Carlo (MCMC) technique to evaluate the *CUE*_0_ and *m*. To get the maximum likelihood estimators of the *CUE*_0_ and *m*, we specified them as the uniform distribution over a set of intervals, which were set as (0, 1) and (−0.1, 0), respectively. The collected CUE values and the corresponding incubation temperatures were used as inversing data to constrain the two parameters. We formally made five parallel runs using the Metropolis-Hastings (M-H) algorithm^45,46^ as the MCMC sampler with 100,000 simulations for each run. Each run started from a random initial point in their respective parameter intervals to eliminate the effect of the initial condition on stochastic sampling. The acceptance rates for the five runs ranged from 5% to 10% which tested by the Gelman-Rubin (G-R) diagnostic method. The initial samples (approximately 1,000 for each run) were discarded after the running means and standard deviations (SDs) were stabilized (regarded as the burn-in period). All the accepted samples from five runs after the burn-in periods (approximately 20,000 samples in total) were used to construct maximum likelihood estimators of both the *CUE*_0_ and *m* (inset of Fig. 3). The evaluation of *CUE*_0_ and *m* were conducted with MATLAB 2016b (The Mathworks, Natick, MA, USA).

### *γ*_*S*_ for different C substrates

***γ***_***S***_ for different C substrates were acquired based on the previous summary by Roels^27^. In all, 26 ***γ***_***S***_ for different C substrate were extracted. According to the classification of Fig. 4, ***γ***_***S***_ in our database were divided into glucose, amino acid, other acid and high-molecular. As ***γ***_***S***_ refers to single substance, there is no accurate ***γ***_***S***_ for the mixed substance in Fig. 4 (i.e., Glucose + N, Glucose + C, Glucose + salt, High-molecular + N, Residue + N, Mixture). ***γ***_***S***_ for residue, H_2_O and inorganic N were still unclear for lack of literature report. The range of *γ*_*S*_ in our database was from 1 to 6. As the most used substrate, glucose owns the degree of reduction of 4. The degree of reduction for amino acid distributed mostly in 3.6 and 5. The distribution of degree of reduction for other acid widely ranged from 1 to 6.

### Statistical analyses

The relationships among CUE, environmental variables and soil properties were quantified by the linear univariate regression analysis. The environmental variables include latitude, longitude, mean annual precipitation (MAP) and mean annual temperature (MAT). The soil properties include soil pH and the ratio of soil carbon to nitrogen content (C/N). We further explored the relationships among CUE, latitude, longitude, temperature, MAP and pH by performing multiple regression analyses. We also performed a Principal Component Analysis (PCA) to examine the relationships among the variables of temperature, soil pH, latitude, longitude and MAP. All statistical analyses and figures (except Fig. 1) were performed in **R** (R 3.3.2, R Development Core Team, 2017).

## Supplementary information

Supplementary materials

Dataset1

## Data Availability

All data generated or analysed during this study are included in this published article (and its Supplementary Information files).
