# Supplementary material for: Global variation of soil microbial carbon-use efficiency in relation to growth temperature and substrate supply
Source: Sci Rep. 2019 Apr 4;9:5621. doi: 10.1038/s41598-019-42145-6 (PMC6449510; doi:10.1038/s41598-019-42145-6)
Supplement: Supplementary file 1 — Supplementary materials [file 41598_2019_42145_MOESM1_ESM.pdf]

Global variation of soil microbial carbon-use efficiency in relation to growth temperature and substrate supply

Yang Qiao[1], Jing Wang[1], Guopeng Liang[2], Zhenggang Du[1], Jian Zhou[1], Chen Zhu[1], Kun Huang[1], Xuhui Zhou[1], Yiqi Luo[2], Liming Yan [1,*], Jianyang Xia[1,*]

[1]Tiantong National Field Observation Station for Forest Ecosystem & Center for Global Change and Ecological Forecasting, School of Ecological and Environmental Sciences, East China Normal University, Shanghai 200062, China

[2]Center for Ecosystem Science and Society, Northern Arizona University, Arizona, Flagstaff, AZ 86011, USA

Correspondence to: Liming Yan

Mailing address: School of Ecological and Environmental Sciences, East China Normal University, 500 Dongchuan Road, Shanghai 200062, China

Email: lmyan@des.ecnu.edu.cn

Supplemental Materials

Table S1. Papers from which the dataset was extracted.

1. Ågren, G.I., Bosatta, E., Magill, A.H., (2001) Combining theory and experiment to understand effects of inorganic nitrogen on litter decomposition. *Oecologia* **128,** 94-98.
2. Anderson, T.-H., Domsch, K.H., (1986) Carbon assimilation and microbial activity in soil. *Zeitschrift für Pflanzenernährung und Bodenkunde* **149,** 457-468.
3. Andreasson F, Bergkvist B, Baath E (2009) Bioavailability of DOC in leachates, soil matrix solutions and soil water extracts from beech forest floors. *Soil Biology & Biochemistry,* **41,** 1652-1658.
4. Behera B, Wagner GH (1974) Microbial Growth Rate in Glucose-Amended Soil1. *Soil Science Society of America Journal,* **38,** 591-594.
5. Blagodatskaya, E., Blagodatsky, S., Anderson, T.-H., Kuzyakov, Y., (2014) Microbial growth and carbon use efficiency in the rhizosphere and root-free soil, *PloS one*, p. e93282.
6. Brant JB, Sulzman EW, Myrold DD (2006) Microbial community utilization of added carbon substrates in response to long-term carbon input manipulation. *Soil Biology & Biochemistry,* **38,** 2219-2232.
7. Bremer EG, Kuikman PJ (1994) Microbial utilization of 14C[U]glucose in soil is affected by the amount and timing of glucose additions. *Soil Biology & Biochemistry,* **26,** 511-517.
8. Chotte, J.L., Ladd, J.N., Amato, M., (1998) Sites of microbial assimilation, and turnover of soluble and particulate 14C-labelled substrates decomposing in a clay soil. *Soil Biology and Biochemistry* **30,** 205-218.

9. Dahlin AS, Witter E (1998) Can the low microbial biomass C-to-organic C ratio in an acid and a metal contaminated soil be explained by differences in the substrate utilization efficiency and maintenance requirements? *Soil Biology & Biochemistry,* **30,** 633-641.

10. Devevre OC, Horwath WR (2000) Decomposition of rice straw and microbial carbon use efficiency under different soil temperatures and moistures. *Soil Biology & Biochemistry,* **32,** 1773-1785.

11. Dijkstra P, Dalder JJ, Selmants PC, Hart SC, Koch GW, Schwartz E, Hungate BA (2011a) Modeling soil metabolic processes using isotopologue pairs of position-specific 13 C-labeled glucose and pyruvate. *Soil Biology & Biochemistry,* **43,** 1848-1857.

12. Dijkstra P, Salpas E, Fairbanks D *et al.* (2015) High carbon use efficiency in soil microbial communities is related to balanced growth, not storage compound synthesis. *Soil Biology & Biochemistry,* **89,** 35-43.

13. Dijkstra P, Thomas SC, Heinrich PL, Koch GW, Schwartz E, Hungate BA (2011b) Effect of temperature on metabolic activity of intact microbial communities: Evidence for altered metabolic pathway activity but not for increased maintenance respiration and reduced carbon use efficiency. *Soil Biology & Biochemistry,* **43,** 2023-2031.

14. Elliott ET, Cole CV, Fairbanks BC, Woods LE, Bryant RJ, Coleman DC (1983) Short-term bacterial growth, nutrient uptake**,** and ATP turnover in sterilized, inoculated and C-amended soil: The influence of N availability. *Soil Biology & Biochemistry,* **15,** 85-91.

15. Farrell M, Hill PW, Wanniarachchi SD, Farrar JF, Bardgett RD, Jones DL (2011) Rapid peptide metabolism: A major component of soil nitrogen cycling? *Global Biogeochemical Cycles,* 25.

16. Farrell, M., Macdonald, L.M., Baldock, J.A., (2015) Biochar differentially affects the cycling and partitioning of low molecular weight carbon in contrasting soils. *Soil Biology and Biochemistry* **80,** 79-88.
17. Fierer, N., Schimel, J.P., (2002) Effects of drying–rewetting frequency on soil carbon and nitrogen transformations. *Soil Biology and Biochemistry* **34,** 777-787.
18. Fisk MC, Fahey TJ (2001) Microbial biomass and nitrogen cycling responses to fertilization and litter removal in young northern hardwood forests. *Biogeochemistry,* **53,** 201-223.
19. Frey SD, Gupta VVSR, Elliott ET, Paustian K (2001) Protozoan grazing affects estimates of carbon utilization efficiency of the soil microbial community. *Soil Biology & Biochemistry,* **33,** 1759-1768.
20. Frey SD, Lee J, Melillo JM, Six J (2013) The temperature response of soil microbial efficiency and its feedback to climate. *Nature Climate Change*, **3,** 395.
21. Gilmour CM, Gilmour JT (1985) Assimilation of carbon by the soil biomass. *Plant and Soil*, **86,** 101-112.
22. Guitong Li, Baogui Zhang, Baoguo Li (2003) Effect of straw pretreatment on soil microbial biomass and respiration activity. *Chi . J. App l. Ecol.* 2003, 14(**12**) : 2225- 2228.
23. Hagerty SB, Van Groenigen KJ, Allison SD et al. (2014) Accelerated microbial turnover but constant growth efficiency with warming in soil. *Nature Climate Change*, **4,** 903.
24. Haiyan Zhu, Yuanjin Bo, Xiangui Lin, Jianguo Zhu, Zubin Xie, Zhihong Cao, Huayong Zhang (2003) Effects of exogenous lanthanum on carbon utilization of soil microorganisms. *Rural Eco-Environment* 19(**1**) : 47- 49
25. Hart SC, Nason GE, Myrold DD, Perry DA (1994) Dynamics of gross nitrogen transformation in an old-growth forest: the carbon connection. *Ecology*, **75,** 880-891.

26. Herron PM, Stark JM, Holt C, Hooker TD, Cardon ZG (2009) Microbial growth efficiencies across a soil moisture gradient assessed using 13 C-acetic acid vapor and 15 N-ammonia gas. *Soil Biology & Biochemistry*, **41,** 1262-1269.

27. Jiang, X., Denef, K., Stewart, C.E., Cotrufo, M.F., (2016) Controls and dynamics of biochar decomposition and soil microbial abundance, composition, and carbon use efficiency during long-term biochar-amended soil incubations. *Biology and Fertility of Soils* **52,** 1-14.

28. Jones DL, Kielland K, Sinclair FL, Dahlgren RA, Newsham KK, Farrar JF, Murphy DV (2009) Soil organic nitrogen mineralization across a global latitudinal gradient. *Global Biogeochemical Cycles*, 23.

29. Kallenbach, C.M., Grandy, A.S., Frey, S.D., Diefendorf, A.F., (2015) Microbial physiology and necromass regulate agricultural soil carbon accumulation. *Soil Biology and Biochemistry* **91,** 279-290.

30. Keiblinger, K.M., Hall, E.K., Wanek, W., Szukics, U., Hämmerle, I., Ellersdorfer, G., Böck, S., Strauss, J., Sterflinger, K., Richter, A., Zechmeister-Boltenstern, S., (2010) The effect of resource quantity and resource stoichiometry on microbial carbon-use-efficiency. *FEMS Microbiology Ecology* **73,** 430-440.

31. Koranda, M., Kaiser, C., Fuchslueger, L., Kitzler, B., Sessitsch, A., Zechmeister-Boltenstern, S., Richter, A., (2013) Seasonal variation in functional properties of microbial communities in beech forest soil. *Soil Biology and Biochemistry* **60,** 95-104.

32. Ladd JN, Jocteurmonrozier L, Amato M (1992) Carbon turnover and nitrogen transformations in an alfisol and vertisol amended with [U-14C] glucose and [15N] ammonium sulfate. *Soil Biology & Biochemistry*, **24,** 359-371.

33. Lee, Z.M., Schmidt, T.M., (2014) Bacterial growth efficiency varies in soils under different land management practices. *Soil Biology and Biochemistry* **69,** 282-290.

34. Lipson, D.A., Monson, R.K., Schmidt, S.K., Weintraub, M.N., (2009) The trade-off between growth rate and yield in microbial communities and the consequences for under-snow soil respiration in a high elevation coniferous forest. *Biogeochemistry* **95,** 23-35.

35. Liu, Y., Tay, J.-H., (2000) Interaction between catabolism and anabolism in the oxidative assimilation of dissolved organic carbon. *Biotechnology Letters* **22,** 1521-1525.

36. Lundberg, P., Ekblad, A., Nilsson, M., (2001) 13C NMR spectroscopy studies of forest soil microbial activity: glucose uptake and fatty acid biosynthesis. *Soil Biology and Biochemistry* **33,** 621-632.

37. Nguyen C, Guckert A (2001) Short-term utilisation of 14 C-[U]glucose by soil microorganisms in relation to carbon availability. *Soil Biology & Biochemistry,* **33,** 53-60.

38. Parsons LL, Smith MS (1989) Microbial Utilization of Carbon-14-Glucose in Aerobic vs. Anaerobic Denitrifying Soils. *Soil Science Society of America Journal,* **53,** 1082-1085.

39. Roberts, P., Jones, D.L., (2012) Microbial and plant uptake of free amino sugars in grassland soils. *Soil Biology and Biochemistry* **49,** 139-149.

40. Santrůckova H, Picek T, Tykva R, Simek M, Pavlů B (2004) Short-term partitioning of 14C-[U]-glucose in the soil microbial pool under varied aeration status. *Biology and Fertility of Soils,* **40,** 386-392.

41. Schimel DS (1988) Calculation of microbial growth efficiency from 15N immobilization. *Biogeochemistry,* **6,** 239-243.

42. Schneckenberger, K., Demin, D., Stahr, K., Kuzyakov, Y., (2008) Microbial utilization and mineralization of [14C]glucose added in six orders of concentration to soil. *Soil Biology and Biochemistry* **40,** 1981-1988.

43. Sharifi, M., Zebarth, B.J., Burton, D.L., Drury, C.F., Grant, C.A., (2013) Mineralization of Carbon-14-Labeled Plant Residues in Conventional Tillage and No-Till Systems. *Soil Science Society of America Journal* **77,** 123-132.

44. Shen, J., Bartha, R., (1996) Metabolic efficiency and turnover of soil microbial communities in biodegradation tests. *Applied and Environmental Microbiology* **62,** 2411-2415.

45. Shields JA, Paul EA, Lowe WE, Parkinson D (1973) Turnover of microbial tissue in soil under field conditions. *Soil Biology & Biochemistry*, **5,** 753-764.

46. Spohn, M., Klaus, K., Wanek, W., Richter, A., (2016a) Microbial carbon use efficiency and biomass turnover times depending on soil depth – Implications for carbon cycling. *Soil Biology and Biochemistry* **96,** 74-81.

47. Spohn, M., Pötsch, E.M., Eichorst, S.A., Woebken, D., Wanek, W., Richter, A., (2016b) Soil microbial carbon use efficiency and biomass turnover in a long-term fertilization experiment in a temperate grassland. *Soil Biology and Biochemistry* **97,** 168-175.

48. Steinweg JM, Plante AF, Conant RT, Paul EA, Tanaka DL (2008) Patterns of substrate utilization during long-term incubations at different temperatures. *Soil Biology & Biochemistry,* **40,** 2722-2728.

49. Sugai SF, Schimel JP (1993) Decomposition and biomass incorporation of 14C-labeled glucose and phenolics in Taiga forest floor : effect of substrate quality, successional state, and season. *Soil Biology & Biochemistry*, **25,** 1379-1389.

50. Tianyu Li, Fengfeng Kang, Hairong Han (2015a) Responses of soil microbial carbon metabolism to the leaf litter composition in Liaohe River Nature Reserve of northern Hebei Province，China. *Chin． J． Appl． Ecol．* 2015，26(**3**) : 715－722．

51. Tianyu Li, Fengfeng Kang, Hairong Han, Jing Gao, Xiaoshuai (2015b) Song Characteristics of Microbial Carbolic Metabolism in Soils of Pinus Tabulaeformis in Liaohe River of Northern Hebei. *Soil* 2015, 47(**3**): 550–557

52. Thiet RK, Frey SD, Six J (2006) Do growth yield efficiencies differ between soil microbial communities differing in fungal:bacterial ratios? Reality check and methodological issues. *Soil Biology & Biochemistry,* **38,** 837-844.

53. Tucker C, Bell J, Pendall E, Ogle K (2013) Does declining carbon - use efficiency explain thermal acclimation of soil respiration with warming? *Global Change Biology,* **19,** 252-263.

54. Van Ginkel JH, Gorissen A, Polci D (2000) Elevated atmospheric carbon dioxide concentration: effects of increased carbon input in a Lolium perenne soil on microorganisms and decomposition. *Soil Biology & Biochemistry,* **32,** 449-456.

55. Van Groenigen KJ, Forristal D, Jones MB, Smyth N, Schwartz E, Hungate BA, Dijkstra P (2013) Using metabolic tracer techniques to assess the impact of tillage and straw management on microbial carbon use efficiency in soil. *Soil Biology & Biochemistry,* **66,** 139-145.

56. Van Hees PaW, Johansson EMV, Jones DL (2008) Dynamics of simple carbon compounds in two forest soils as revealed by soil solution concentrations and biodegradation kinetics. *Plant and Soil,* **310,** 11-23.

Table S2. List of data source experiments.

No.	Incubation temperature (ºC)	Substrate addition	Incubation time (d)	Calculate method	Data location	Mean CUE ±SD	Reference number
1	not mention	litter	>30	Directly extracted	Table1	0.22±0.03	1
2	22	glucose	1-7	Directly extracted	Table2	0.47±0.07	2
3	20	soil leachate	not mention	Directly extracted	Fig. 2	0.41±0.17	3
4	25	glucose	not mention	Directly extracted	article	0.39	4
5	22	glucose	1-7	Directly extracted	Table3	0.30±0.10	5
6	22	glucose, glutamate, oxalate, phenol	1-7	Directly extracted	Table2	0.38±0.28	6
7	21	glucose	>30	Eq. 4	Fig.1, Fig.2	0.20±0.10	7
8	25	glucose, starch, legume, wheat	>30	Directly extracted	Table4	0.41±0.19	8
9	28.5	glucose	7-30	Eq. 4	Table3, Table4, Table5	0.18±0.06	9
10	5, 15, 25	rice straw	not mention	Directly extracted	Table3	0.44±0.11	10
11	20	glucose, sodium, pyruvate	not mention	Directly extracted	Table2	0.72±0.01	11
12	21	glycogen, synthesis,	not mention	Directly extracted	Table1	0.71±0.15	12
13	4, 20	glucose, pyruvate	not mention	Directly extracted	Table3	0.74±0.01	13
14	25	glucose, $(NH_4)_2SO_4$	1-7	Directly extracted	Table3, Table4	0.48±0.13	14
15	10	alanine, dialanine, trialanine	not mention	Directly extracted	Table5, Fig.3	0.72±0.11	15

16	22	glucose, fructose, sucrose, oxalate, phenylalanine	7	Directly extracted	Fig.5	0.76±0.19	16
17	20	glucose, glutamic	1	Eq. 4	Fig.9	0.49±0.14	17
18	22	not mention	not mention	Directly extracted	Table6	0.28±0.06	18
19	25	glucose	<1d	Directly extracted	Table5	0.52±0.12	19
20	5, 15, 25	glucose, glutamic, phenol, oxalic acid	not mention	Directly extracted	Fig.1, Fig.2	0.29±0.22	20
21	24	glucose, NH_4NO_3, litter	1-7	Directly extracted	Table2	0.52±0.19	21
22	25	litter, $(NH_4)_2SO_4$	>30	Eq. 5	Fig.1, Fig.3	0.15±0.15	22
23	5, 10, 15, 20	glucose, pyruvate	not mention	Directly extracted	Fig.1	0.72±0.01	23
24	25	glucose, $LaCl_3$	1-7	Eq. 4	Table2	0.63±0.04	24
25	25	not mention	>30	Directly extracted	Fig.7	0.38±0.19	25
26	not mention	acetic acid, NH_3	not mention	Directly extracted	Fig.5	0.50±0.11	26
27	25	glucose	<1	Directly extracted	Fig.5	0.58±0.06	27
28	20	not mention	not mention	Directly extracted	Fig.4	0.71±0.05	28
29	25	glutamic acid	<1	Directly extracted	Fig.1	0.33±0.15	29
30	22	glucose	<1	Directly extracted	Fig.3	0.48±0.10	30
31	12	glucose, protein, cellulose, cell walls	1-7	Directly extracted	Fig.3b	0.30±0.10	31

32	25	glucose, $(NH_4)_2SO_4$	>30	Directly extracted	Table2	0.62±0.17	32
33	25	leucine	1	Directly extracted	Fig.1, Fig.2, [illegible]	0.38±0.08	33
34	14	glucose	not mention	Directly extracted	Fig.2	0.45±0.04	34
35	not mention	glucose, fructose, sucrose, lactose, xylose, glycine, alanine, glutamic acid, phenylalanine, acetic acid, propionic acid, butyric acid, butanol, benzoic acid	not mention	Directly extracted	Table1	0.63±0.14	35
36	25	glucose	1-7	Directly extracted	Fig.7	0.67±0.10	36
37	22	glucose	1-7	Directly extracted	Table2	0.81±0.13	37
38	not mention	glucose	1-7	Directly extracted	Fig.1	0.57±0.10	38
39	1, 5, 10, 15	glucose, glucosamine	1	Directly extracted	Fig.2	0.94±0.03	39
40	not mention	glucose	1-7	Directly extracted	Table3	0.64±0.13	40
41	25	$(NH_4)_2SO_4$	not mention	Directly extracted	Table3	0.41±0.09	41
42	18	glucose	7-30	Eq. 4	Fig.4	0.54±0.18	42
43	25	wheat residues	>30	Directly extracted	Fig.3	0.91±0.01	43
44	27	glucose	7-30	Directly extracted	article	0.57±0.13	44
45	not mention	glucose	>30	Eq. 3	Table1, Table2	0.35±0.25	45
46	15	H_2O	1	Directly extracted	Fig.4	0.24±0.08	46

47	15	H_2O	1	Directly extracted	Fig.3	0.38±0.06	47
48	15, 25	cellobiose	>30	Directly extracted	Table1	0.70±0.07	48
49	11	glucose, benzoic acid,	1-7	Directly extracted	article	0.55±0.23	49
50	25	litter	>30	Eq. 5	Fig.1, Fig.2	0.18±0.10	50
51	25	not mention	>30	Eq. 5	Fig.1, Fig.2	0.09±0.02	51
52	25	glucose	<1	Directly extracted	Table1	0.67±0.07	52
53	1.5, 10, 22.5	dextrose	1-7	Directly extracted	Fig.8	0.76±0.14	53
54	14, 16	residue	not mention	Eq. 4	Fig.2	0.31±0.11	54
55	20	glucose, pyruvate	not mention	Directly extracted	Fig.5	0.77±0.01	55
56	5	glucose	7-30	Directly extracted	Fig.1	0.63±0.12	56

Table S3. Coefficients of variables based on the multiple stepwise regression analysis.

	Unstandardized Coefficients		Standardized Coefficients	t	Sig
	B	Std. Error	Beta		
Constant	0.319	0.057	-	5.557	0.000
Temperature	-0.025	0.002	-0.551	-13.952	0.000
Glucose	0.172	0.019	0.306	8.845	0.000
Latitude	-0.006	0.001	-0.454	-11.609	0.000
Longitude	-0.001	0.000	-0.327	-9.195	0.000
MAP	0.000	0.000	0.259	7.065	0.000
pH	0.032	0.008	0.138	3.825	0.000
Organic acid	-	-	-	-0.492	0.623
Plant residue	-	-	-	1.929	0.054
Inorganic substrate	-	-	-	-1.731	0.084

Table S4. CUE formula including all environmental factors (i.e., Temperature, Latitude, Substrate type, Longitude, MAP, and pH) based on different substrate type.

Substrate type	CUE formula	R^2
Glucose	CUE=0.491-0.025×(T-20)-0.006×Latitude-0.001×Longitude+0.0002×MAP+0.032×pH	0.612
Other	CUE=0.319-0.025×(T-20)-0.006×Latitude-0.001×Longitude+0.0002×MAP+0.032×pH	0.612

Figure S1. The CUE changes with latitude (a), longitude (b), mean annual temperature (MAT, c), mean annual precipitation (MAP, d), soil pH (e) and the ratio of soil carbon to nitrogen content (soil C/N, f) of the given site.

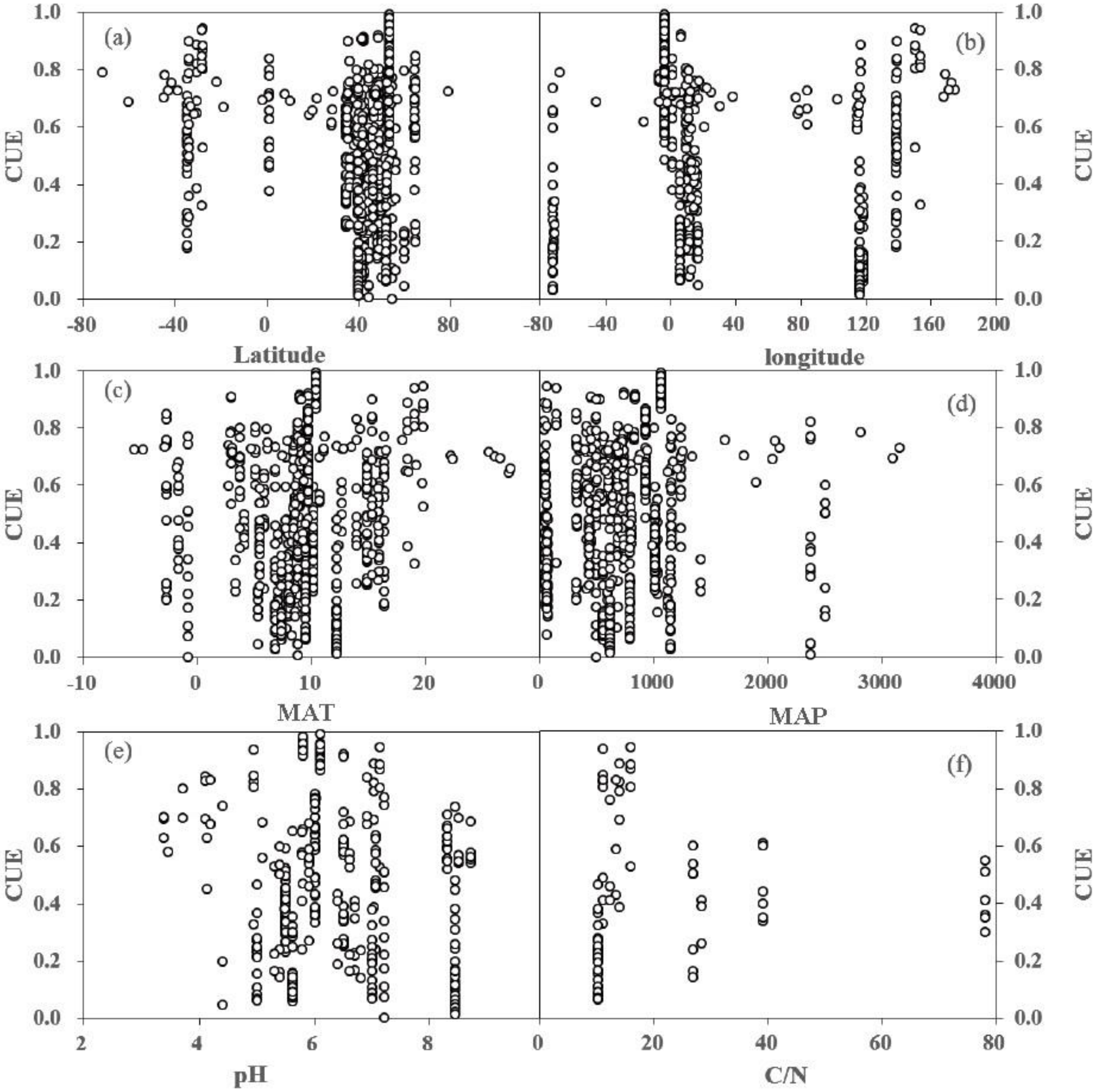

Figure S2. Principal Component Analysis (PCA) for all the single factor (Latitude, Longitude, Temperature, MAP, pH).

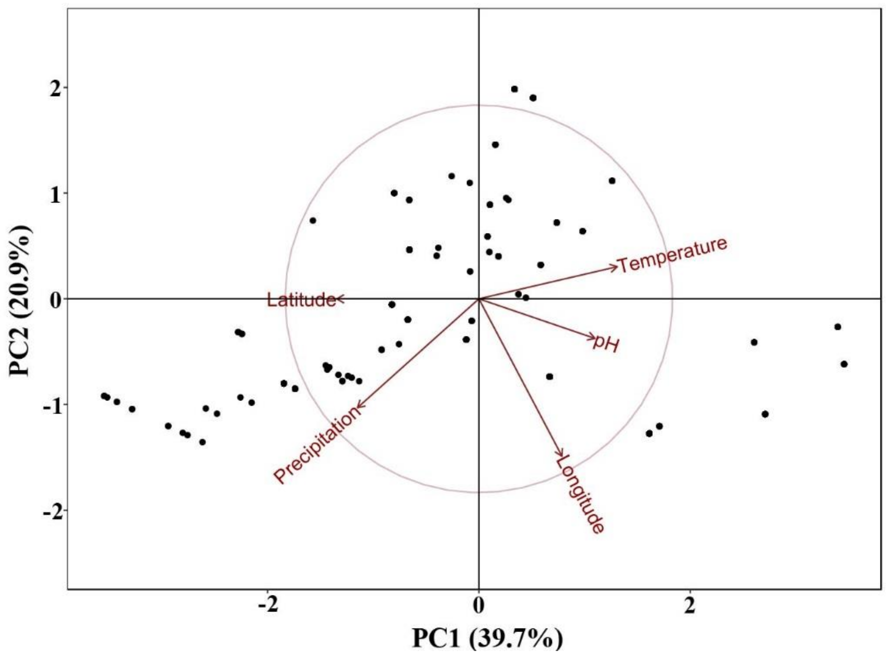

Figure S3. PRISMA flow-diagram of literature search and study selection.

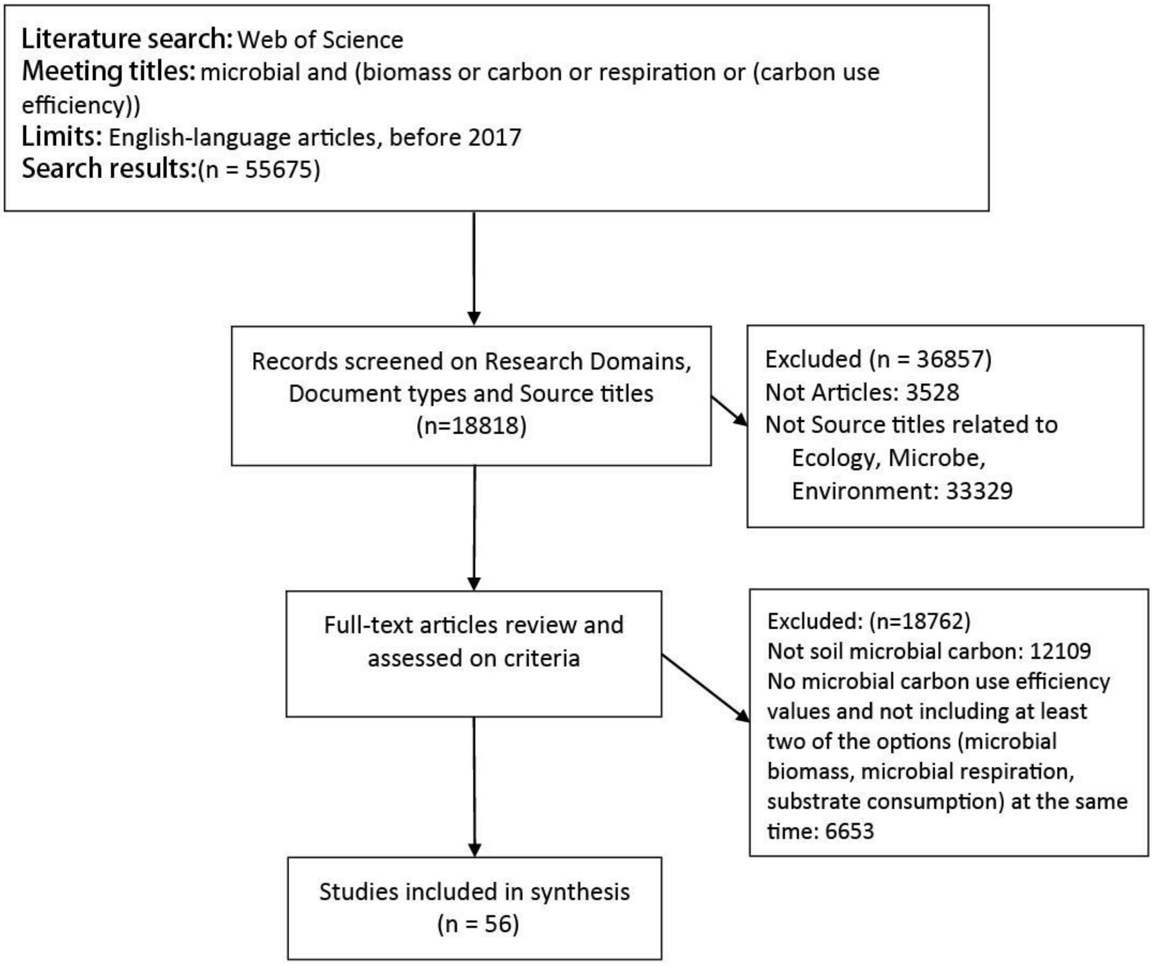

MCMC code for evaluation of CUE_0 and *m*.

```
clear all;
close all;
format long e;

mydata=xlsread('cue2.xlsx');
T = mydata(:,1);
CUE = mydata(:,2);
C = [0.31;-0.016;18];
Cmin = [0;-0.1;10];
Cmax = [1;0;30];

Diff = Cmax-Cmin;
L_P = length(C);
DJ = 2*var(CUE);
J_last = 30000;
upgraded = 0;
nsimu = 1000000;

for simu = 1:nsimu
counter = simu;
upgraded = upgraded;
        while true
                Cnew = C + (rand(3,1)-0.5).*Diff;
                Logic = Cnew>Cmin&Cnew<Cmax;
                  if sum(Logic) == L_P
                          break;
                  end
        end

        CUEsimu = Cnew(1) + Cnew(2).*(T-Cnew(3));
        J = (norm(CUE-CUEsimu))^2;
        J_new = J/DJ;
        %J_new    = J/DJ*200;             %for every year
        delta_J = J_new-J_last;
              if min(1,exp(-delta_J)) >rand
                  C_op = Cnew;
                  J_last = J_new;
                  upgraded = upgraded + 1;
                  C_upgraded(:,upgraded) = C_op;
                  J_upgraded(:,upgraded) = J_last;
              end
end

C_upgraded = C_upgraded';
```
